# Factors affecting the rumen fluid foaming performance in goat fed high concentrate diet

**DOI:** 10.3389/fvets.2024.1299404

**Published:** 2024-02-16

**Authors:** Zehao Tan, JunFeng Liu, Lizhi Wang

**Affiliations:** ^1^Animal Nutrition Institute, Sichuan Agricultural University, Chengdu, China; ^2^Wuhan Xinzhou Vocational High School, Wuhan, China

**Keywords:** goat, high concentrate diet, rumen bloat, foam, protein

## Abstract

Feeding high concentrate diets is highly prone to rumen bloat in ruminants, which is very common in production. This study explored the factors responsible for the occurrence of foamy rumen bloat. The experiment was conducted using goats as test animals, fed high concentrate diets and scored for rumen distension into high, medium and low bloat score groups. Rumen fluid was collected from 6 goats in each group separately. The foaming production, foam persistence, pH value, viscosity and the content of protein, total saccharide and mineral elements in rumen original fluid (ROL) were measured, and the protein and total saccharide content in rumen foam liquid (RFL) and rumen residual liquid (RRL) were determined. The results showed that the protein content in rumen original fluid and rumen foam liquid was significantly higher than that in rumen residual liquid (*p* < 0.05), and the protein content in rumen foam liquid was 10.81% higher than that in rumen original fluid. The higher the rumen bloat score, the higher the foam production, foam persistence, viscosity, protein, Ni, Mg, Ca, and K concentrations of the rumen original fluid, and the lower the PH and Na concentrations of the rumen original fluid; correlation analysis showed that the viscosity of the rumen original fluid was significantly and positively correlated with the foam production and foam persistence (*p* < 0.05). Foaming production and foam persistence of rumen original fluid were significantly and positively correlated with the contents of protein, total saccharide, K, Ca, Mg and Ni (*p* < 0.05). and negatively correlated with the content of Na (*p* < 0.05); after controlling other components those were significantly related to the foaming performance of rumen original fluid only protein still was significantly positively correlated with the foam persistence of rumen original fluid (*P*<0.05). In summary, the contents of protein, total saccharide and mineral elements in the rumen fluid had a significant effect on the foaming performance of rumen in ruminants, with protein playing a decisive role and the other components playing a supporting role. Reducing the content of protein in the diet in production is beneficial to reduce the occurrence of rumen bloat in ruminants.

## 1 Introduction

In the modern intensive farming model, the use of high concentrate diet (HCD) is often increased to improve production performance (1, 2). Horse used for meat production are often fed a starch-based concentrate feed in many European countries to shorten the fattening period. However, long-term feeding HCD tends to increase the total amount of volatile fatty acids and valeric acid in the horse’s intestinal tract, which reduces the integrity of the intestinal mucosa, thus leading to gastrointestinal inflammation (3, 4). Offering total mixed rations (TMR) to ruminants, promote synchronized intake of concentrate and roughage can reduce the risk of rumen toxicity and promote animal health and welfare (5). However, long-term feeding of HCD can lead to digestive diseases, rumen bloat is one of the common digestive diseases. One study found that digestive-related mortality on rangelands accounted for 19.5–28.4% of all mortality compared to mortality from other causes, with 96.3% of digestive mortality diagnosed as rumen bloat (6). In most cases, rumen bloat leads to the death of ruminants due to digestive diseases. Early studies believed that too much and too fast gas produced by rumen fermentation was the main reason of rumen bloat induced by HCD. Rumen microorganisms decompose starch to produce low-grade fatty acids, carbon dioxide and methane (7), and use monosaccharides and disaccharides produced by feed decomposition to synthesize glycogen for storage in microorganisms. Therefore, compared with fiber substances, equivalent amount of starch produces more gas in the rumen, so the rumen is prone to bloat (8). The starch content in ruminants rises in accordance with the increased proportion of concentrate in their diet, leading to an elevated incidence of rumen bloat. However, studies have found that although the digestion rate and gas production rate of pressed barley in the rumen are faster than that of whole barley, the incidence of rumen bloat of pressed barley diet is significantly lower than that of whole barley diet (9, 10). In addition, studies have shown that rumen fermentation rate and degree of wheat is higher than that of barley, sorghum or corn (11). However, recent studies reported that rumen bloat is caused by foam-encased gases produced by rumen fermentation that cannot be properly expelled (12–14). Protein (15), polysaccharide (16), mineral ions (17), etc. coming from diet, rumen microbial synthesis and rumen microbial fermentation diet in rumen fluid may act as the foaming agent or foam stabilizer. But up to now, the key components in rumen fluid that affect the foaming production or foam persistence have not been determined. Our study hypothesized that factors affecting rumen foam production and foam stability may be related to the nutrient composition in high concentrate. In this study, the components affecting the foaming performance of rumen fluid in goats fed with HCD were analyzed. The results can lay a foundation for the rational application of HCD in production.

## 2 Materials and methods

For The research procedure used in current study was approved by Animals policy and welfare committee of Agricultural research organization of Sichuan province China and in agreement with rules of the Animal Care and Ethical Committee of the Sichuan Agricultural University (Ethics Approval Code: SCAUAC201608-5).

### 2.1 Experimental animals and management

The animal experiments were conducted at the farm of the Animal Nutrition Institute, Sichuan Agricultural University, Ya’an, Sichuan Province, China. The experimental animals were 26 healthy Jianzhou big-eared goats, aged 8–10 months, weighing about 30 kg, which were immunized and dewormed before the experiment. During the experiment, the goats were kept in a single pen and feed twice a day (feeding at 9:00 in the morning and at 17:00 in the afternoon, respectively) to ensure that the goats eat freely, and enable the goats to drink freely.

### 2.2 Experimental diet

The experimental diet were configured in according with the to the Chinese Feeding Standard of Meat-producing Sheep and Goats (NY/T816-2004). Before the start of the experiment, the goats were fed with oat hay, and the diet was gradually transitioned to a total mixed ration with a concentrate-roughage ratio of 80:20 in 14 days (see Table 1 for the composition and nutrient level of the diet).

**Table 1:** Composition and nutrient levels of experimental diet (basis on DM).

Ingredients	%	Nutrients	%
Corn	48.25	CP	15.93
Wheat bran	7.30	EE	5.23
Soybean meal	10.50	NDF	19.55
Rapeseed meal	2.00	ADF	7.34
Cottonseed cake	8.00	Ca	0.76
Oat grass	4.00	P	0.49
Wheat straw	2.00	Starch	36.18
Alfalfa hay	14.00	DE (Mcal.kg^−1^)	2.88
Calcium carbonate	1.00		
Calcium hydrophosphate	0.45		
NaCl	0.50		
NaHCO_3_	1.00		
Premix^1^	1.00		
Total	100.00		

^1^The premix provided the following per kg of diet: vitamin A 2200 IU; vitamin D 250 IU; vitamin E 20 IU; Fe 40 mg; Cu 10 mg; Zn 30 mg; Mn 40 mg; I 0.8 mg; Se 0.2 mg; Co 0.11 mg.

### 2.3 Chemical analysis

Chemical analysis was carried out according to the methodology described in the literature (18, 19): Diet samples were dried in an oven at 65°C and passed through a 1-mm sieve before determining the DM was determined. Crude protein (CP) content was determined by Kjeldahl method. Ether extract (EE) was determined by Soxhlet extraction method as described by AOAC (20). Ash was determined by scorching in a muffle furnace at 550°C. Neutral detergent fiber (NDF) and acid detergent fiber (ADF) were determined using the method of Van Soest et al. (21). In order to avoid the interference of aflatoxin (AFB1) on the experimental results, the content of aflatoxin in feed ingredients was effectively controlled in this study. Control of aflatoxins in feed at levels well below the established safety limits for animal feed. This precaution was taken to safeguard the health and welfare of the animals. Aflatoxin contamination in animal feed can cause serious health hazards, including effects on growth and damage to the liver by maintaining feed quality within safe limits (22), we aim to minimize the potential impact of aflatoxins on the results of research.

### 2.4 Bloat scoring and sample collection

A 20-day feeding trial was performed. The 14th-19th days of the feeding trial, animals were scored for bloat 2-3 h after morning feeding daily, three raters scored the animals’ bloat on a 0–5 severity scale (From 0 to 5, the severity of bloat increased): 0 = no foam; 1 = slight foam, but no pressure and abdominal bloat; 2 = some foam, enough pressure to expel foam, but no abdominal bloat; 3 = some foam, enough pressure to cause abdominal bloat on one side; 4 = some foam, enough pressure to cause abdominal bloat on right and left sides; 5 = some foam, severe abdominal bloat, and in a state of severe compression (23). The average of the three scorers’ scores was taken as the bloat score for each goat. The mean and standard deviation (SD) of bloat score (BS) was calculated for each goat. Then, the SD values above and below the mean were used to group animals into high-bloat-score (HBS, BS > mean + 0.5 × SD) group, middle-bloat-score (MBS, mean 0.5 × SD < BS < mean + 0.5 × SD) group and low-bloat-score (LBS, BS < mean – 0.5 × SD) group according to the literature described method (24, 25). Rumen contents was collected from six goat in each group separately after 3 h after the 20th day morning feeding: The selected goats were immobilized and the rumen contents were collected using a stomach tube with a vacuum pump, the first 30 mL of contents pumped were discarded. Afterwards, 50 mL of rumen contents were collected and the collected samples were photographed and observed. The pH value was immediately measured using a portable magnetic-thunder pH meter (PHBJ-260, China), were filtered through four layers of gauze to obtain the rumen fluid and after centrifugation (3,000 g, 2 min), the rumen supernatant was collected and defined as the rumen original liquid (ROL) in this experiment. The ROL was stored at - 20°C for subsequent analysis.

### 2.5 Determination of foaming performance

The improved Roche method combined with the Rudin method was used to measure the foamability and foam stability of ROL (14, 26). The foaming power measured by the improved Roche method is expressed in terms of the volume of foam obtained under specific experimental conditions. The ROL was first treated in a constant temperature water bath at 39°C for half an hour, introduce 30 mL of ROL into a 100 mL airtight separatory funnel tube, slowly inject CO2 gas into the ROL, transforms ROL into a massive foam and continue ventilation at a pressure of 1 Pascal (Pa) for 60 s. Foaming power is related to the decay of foam volume within 5 min after foam formation. At the end of inflation, record the number of milliliters of foam formed at 30 s, 1 min, and 5 min after stopping the liquid flow, and the average value of the three time points is taken as the foaming production (mL) of the ROL. Open the sampling valve of the separatory funnel to let the rumen fluid flow out slowly, and collect it with a 50 mL beaker. When the foam is about to flow out, close the sampling valve immediately, and the fluid collected at this time is defined as rumen residual liquid (RRL). The Rudin method measures foam stability with attention to the effects of temperature, ventilation rate, gas type, and other factors. After ventilating to convert ROL to foam, The time required for the foam column to collapse by itself until all the foam completely disappears is used as an indicator of foam stability, expressed in foam persistence (min), each sample was measured 3 times and the average value was taken. The liquid formed after the collapse of the foam column was collected and defined as rumen foam liquid (RFL).

### 2.6 Determination of protein content

Coomassie blue staining method (14) was used to determine the protein content in the ROL, RRL and RFL, respectively. The brief process is: take 1 mL of liquid, centrifuge (10,000 g, 10 min) and take the supernatant and dilute it with normal saline at a ratio of 1:3. Dilute the Coomassie Brilliant Blue storage solution with distilled water at a ratio of 1:4. Add 1.5 mL of diluted Coomassie Brilliant Blue working solution to 25 μL of sample supernatant., shake and mix. After standing at room temperature for 10 min, the OD595nm value was measured under a microplate reader.

### 2.7 Determination of viscosity

The dynamic viscosity of ROL was determined by capillary viscometer (GB/T 22235–2008) (27). The process is: take 5 mL of ROL and add it to the inlet of the viscometer, and let it stand in a water bath at (39 ± 0.1) °C for 5 min. Use the ear washing ball to suck the liquid, and let the liquid flow down naturally under the action of gravity after the rumen fluid is sucked up to the scale line. Use a stopwatch to record the time t1 when the ROL flows through the upper and lower scale lines of the viscometer, and repeat 3 times to get the average value. Finally, take 5 mL of absolute ethanol and repeat the above steps to obtain the time t2, and finally calculate the liquid viscosity according to the formula.

### 2.8 Determination of saccharide content

The phenol-sulfuric acid method (14) was used to measure the total saccharide content in ROL, RRL and RFL. The process is: take the glucose standard solution and configure the concentration of the standard curve according to the instructions. Take 1 mL of liquid, centrifuge for 10 min to get supernatant, dilute 1,000 times with normal saline for later use. Take 2 mL of the standard substance of each concentration and the sample to be tested, add 1 mL of the pre-prepared 5% phenol solution, shake and mix. Slowly add 5 mL of concentrated sulfuric acid along the tube wall, shake well, and let stand at room temperature for 30 min. A microplate reader (SpectraMax-190, Molecular Devices, United States) was used to measure the OD490nm value of the standard and samples, and calculate the total saccharide concentration of the samples.

### 2.9 Determination of mineral element content

The concentrations of main mineral elements (Na, K, Ca, Mg, S, P, Cl) and trace mineral elements (Fe, Cu, Mn, Zn, Ni) in ROL were determined by an Agilent Technologies 7500c inductively coupled plasma mass spectrometry (28) (ICP-Ms) system (Agilent Technologies, Santa Clara, CA). According to the test results, the anion and cation balance value (CAD), the sum of the concentration of cations (Na, K, Ca, Mg, Fe, Cu, Mn, Zn, Ni) and the sum of the concentration of anions (S, P, Cl) was calculated, respectively. The CAD unit is mEq/Kg, and the calculation formula is CAD = (Na/23+ K/39) - (Cl/35 + S/16) (29).

### 2.10 Calculations and statistical analyses

All data were first preliminarily organized using Excel 2016. Using SPSS 23.0 statistical software, the Shapiro–Wilk test and Levene’s test were first performed for normality and chi-square test (30). A one-way ANOVA was performed on the differences between groups. Duncan’s multiple comparisons were then performed. And the Pearson method in SPSS 23.0 was used to perform correlation analysis and partial correlation analysis between foaming performance and components of rumen original fluid. The differences were considered statistically significant when *p* < 0.05. All data were presented as mean ± standard deviation.

## 3 Results

### 3.1 Comparison of foaming characteristics

Compared to the LBS group,the HBS and MBS groups exhibited significantly higher foaming production,and viscosity and significantly lower PH values. Moreover, the HBS group demonstrated notably higher foaming production, pH values was significantly lower than that of the MBS group. Additionally, the foam persistence of the LBS group was significantly lower than both the HBS and MBS groups (*p* < 0.05) (Table 2).

**Table 2:** The foaming performance, pH value and viscosity of rumen original liquid (*n* = 6).

Items	Group^1^		
	LBS^2^	MBS^2^	HBS^2^
Foaming production (mL)	11.97 ± 3.33^c^	17.31 ± 0.70^b^	23.76 ± 4.60^a^
Foam persistence (min)	15.45 ± 7.78^b^	29.91 ± 5.53^a^	32.76 ± 5.96^a^
pH value	6.36 ± 0.06^c^	6.16 ± 0.03^b^	6.00 ± 0.16^a^
Viscosity (mpa.s)	52.74 ± 5.66^c^	73.30 ± 8.48^b^	97.67 ± 11.09^a^

^1^In the same row, values with same or no small letter superscripts meaning no significant difference (*p* > 0.05), at the same time different small letter superscripts meaning significant difference (*p* < 0.05). ^2^LBS, low rumen bloat score; MBS, medium rumen bloat score; HBS, high rumen bloat score.

### 3.2 Comparison of protein and total saccharide content

The protein content in the ROL, the difference among the three groups all reached a significant level (*P* < 0.05), and the higher the bloat score, the higher the protein content in the ROL (Table 3). The protein content in the RFL was significantly lower in the MBS group and in the LBS group than in the HBS group (*P* < 0.05), but there was no significant difference between the MBS group and the LBS group (*P* > 0.05). The protein content in RRL had no significant difference among the three groups (*P* > 0.05). The total saccharide content in ROL, RFL and RRL was significantly lower in MBS group and LBS group than in HBS group (*P* < 0.05), but there was no significant difference between MBS group and LBS group (*P* > 0.05).

**Table 3:** The comparison of protein and total saccharide content in the rumen liquid among groups (*n* = 6).

Items	Group^1^		
	LBS^2^	MBS^2^	HBS^2^
Protein content (g/L)			
Rumen original liquid	4.4 ± 0.47^c^	5.15 ± 0.35^b^	6.25 ± 0.69^a^
Rumen foam liquid	5.09 ± 0.41^b^	5.66 ± 0.37^b^	6.78 ± 0.65^a^
Rumen residual liquid	2.97 ± 0.43	2.87 ± 0.78	3.43 ± 0.97
Total saccharide content (g/L)			
Rumen original liquid	5.51 ± 0.99^b^	8.75 ± 2.17^b^	18.14 ± 4.88^a^
Rumen foam liquid	5.65 ± 0.99^b^	8.74 ± 1.9^b^	17.97 ± 5.08^a^
Rumen residual liquid	5.33 ± 0.74^b^	8.43 ± 1.88^b^	18.05 ± 4.85^a^

^1^In the same row, values with same or no small letter superscripts meaning no significant difference (*P* > 0.05), at the same time different small letter superscripts meaning significant difference (*P* < 0.05). ^2^LBS, low rumen bloat score; MBS, medium rumen bloat score; HBS, high rumen bloat score.

Regardless of grouping effect, the comparison of protein and total saccharide content among ROL, RRL and RFL was presented in Table 4. The protein content, ROL and RFL were significantly higher than that of RRL (*P* < 0.05), although the difference between ROL and RFL was not significant (*P* > 0.05), but in value, RFL was 10.81% higher than ROL. There was no significant difference in total saccharide content among ROL, RRL and RFL (*P* > 0.05) (Table 4).

**Table 4:** The comparison of protein and total saccharide content among rumen original liquid, rumen foam liquid and rumen residual liquid (*n* = 18).

Items	Protein content (g/L)^1^	Total saccharide content (g/L)^1^
Rumen original liquid	5.27 ± 0.92^a,1^	10.80 ± 6.26
Rumen foam liquid	5.84 ± 0.86^a^	10.79 ± 6.16
Rumen residual liquid	3.09 ± 0.75^b^	10.61 ± 6.27

^1^The same column of data with the same or no letter indicates that the difference is not significant (*p* > 0.05), and the same column of data with different shoulder letters indicates that the difference is significant (*p* < 0.05).

### 3.3 Foaming performance and content of mineral elements

The content of Ni in HBS group and MBS group was significantly higher than that in LBS group (*P* < 0.05); the content of Na in HBS group and MBS group was significantly lower than that in LBS group (*P* < 0.05); The content of Ca in the HBS group was significantly higher than that in the MBS group and the LBS group (*P* < 0.05); the content of Mg in the HBS group and the MBS group significantly higher than the LBS group (*P* < 0.05); the sum of the cation concentrations in the HBS group and the MBS group was significantly higher than that in the LBS group (*P* < 0.05), and the sum of the cation concentrations in the HBS group was significantly higher than that in the MBS group (*P* < 0.05) (Table 5).

**Table 5:** Contents of mineral elements in rumen fluid (*n* = 6).

Item	Group		
	LBS^2^	MBS^2^	HBS^2^
Fe (mg/Kg)	40.24 ± 10.27^1^	32.98 ± 6.92	44.84 ± 11.63
Cu (mg/Kg)	2.208 ± 0.67	1.596 ± 0.41	2.26 ± 0.29
Mn (mg/Kg)	16.7 ± 4.20	14.72 ± 2.61	15.56 ± 4.51
Zn (mg/Kg)	1.25 ± 0.38	0.906 ± 0.08	1.304 ± 0.35
Ni (mg/Kg)	0.154 ± 0.05^b^	0.236 ± 0.02^a^	0.28 ± 0.08^a^
Na (mg/Kg)	3,484 ± 41.59^b^	3,482 ± 8.36^a^	3,362 ± 61.81^a^
K (mg/Kg)	2090 ± 22.36^b^	2,142 ± 13.04^b^	2,272 ± 69.79^a^
Ca (mg/Kg)	351.2 ± 93.22^b^	418 ± 50.77^b^	509.2 ± 24.68^a^
Mg (mg/Kg)	78.84 ± 20.69^b^	136.6 ± 17.99^a^	151.8 ± 40.75^a^
S (mg/Kg)	306.4 ± 98.54	235.2 ± 22.84	278.6 ± 45.44
P (mg/Kg)	1966 ± 243.88	1940 ± 266.08	1984 ± 291.08
Cl (mg/Kg)	286.89 ± 53.76	297.16 ± 58.87	316.48 ± 63.37
CAD (mEq/Kg)	4980.71 ± 132.34	5091.64 ± 50.99	5038.92 ± 88.53
Sum of Cation (mg/Kg)	490.59 ± 119.55^c^	605.04 ± 65.78^b^	725.24 ± 38.31^a^
Sum of Anion (mg/Kg)	8133.29 ± 311.57	8096.36 ± 287.72	8213.08 ± 323.19

^1^In the same row, values with same or no small letter superscripts meaning no significant difference (*P* > 0.05), at the same time different small letter superscripts meaning significant difference (*P* < 0.05). ^2^LBS, low rumen bloat score; MBS, medium rumen bloat score; HBS, high rumen bloat score.

### 3.4 Correlation analysis between foam properties and rumen fluid properties

The pH value of rumen fluid was significantly negatively correlated with its foaming production and foam persistence (*P* < 0.05), and the viscosity was significantly positively correlated with foaming production and foam persistence (*P* < 0.05) (Table 6).

**Table 6:** Correlation analysis of pH value and viscosity with foaming production and foam persistence of rumen fluid (*n* = 18).

Items	Foaming production (mL)	Foam persistence (min)
pH value	−0.70^** 1^	−0.82^**^
Viscosity (mpa.s)	0.96^**^	0.76^**^

^1^Pearson correlation analysis was used for correlation analysis, *P<0.05, **P<0.01.

### 3.5 Correlation analysis of foaming performance and components

There was a significant positive correlation between the protein concentration in the ROL and the foaming production and foam persistence of ROL (*P* < 0.05), which were also significant positive correlation (*P* < 0.05) with the total saccharide concentration of rumen fluid (Table 7).

**Table 7:** Correlation analysis between foaming performance and components of rumen original fluid (*n* = 18).

Items	Foaming performance of rumen original fluid	
	Foaming production (mL)	Foam persistence (min)
Protein (g/L)	0.90^**^	0.79^**^
Total saccharide (g/)	0.73^**^	0.56*
Fe (mg/Kg)	0.36	0.19
Cu (mg/Kg)	0.24	0.01
Mn (mg/Kg)	0.17	−0.16
Zn (mg/Kg)	0.38	0.04
Ni (mg/Kg)	0.81^**^	0.58^*^
Na (mg/Kg)	−0.90^**^	−0.52^*^
K (mg/Kg)	0.96^**^	0.62^*^
Ca (mg/Kg)	0.88^**^	0.68^**^
Mg (mg/Kg)	0.55^*^	0.76^**^
S (mg/Kg)	0.15	−0.13
P (mg/Kg)	0.17	0.24
Cl (mg/Kg)	0.40	0.3
CAD 2 (mEq/Kg)	−0.467	−0.168
The sum of the cations (mg/Kg)	0.84^**^	0.75^**^
The sum of the anions (mg/Kg)	0.33	0.29

^1^Pearson correlation analysis was used for correlation analysis, *P<0.05, **P<0.01.2. CAD, anion and cation balance value.

The content of Ni in trace mineral elements was significantly positively correlated with the foaming production and foam persistence of rumen fluid (*P* < 0.05); the content of Na in main mineral elements was significantly Negatively correlated with the foaming production and foam persistence of rumen fluid (*P* < 0.05); the content of K, Ca, Mg in the main mineral elements and the foaming production and foam persistence of the rumen fluid were significantly positively correlated (*P* < 0.05); There was a significant positive correlation between the sum of cations and the foam production and foam persistence of rumen fluid (*P* < 0.05) (Table 7).

### 3.6 Analysis of partial correlation between foaming production performance and rumen fluid components

Correlation analysis was performed on the components in Table 7 those were significantly correlated with the foaming performance of the rumen fluid. The results showed that after controlling the influence of other components, only the protein and the foam persistence was significantly positively correlated (*p* < 0.05). The correlation between other components and rumen foaming production and foam persistence, and the correlation between protein and foaming production were no longer significant (*p* > 0.05). And compared with the correlation analysis, the correlation between the protein and the foam persistence in the partial correlation analysis decreased (Table 8).

**Table 8:** Partial correlation analysis between foaming performance and components of rumen fluid.

Items	Foaming performance of rumen fluid	
	Foaming production (mL)	Foam persistence (min)
Protein (g/L)	0.49	0.75^*^
Total saccharide (g/L)	−0.69	−0.33
Ni (mg/Kg)	0.47	0.10
Na (mg/Kg)	−0.51	−0.45
K (mg/Kg)	0.43	−0.56
Ca (mg/Kg)	0.63	0.52
Mg (mg/Kg)	0.58	0.62
The sum of the cations (mg/Kg)	−0.55	0.50

^1^Pearson correlation analysis was used for correlation analysis, *P<0.05, **P<0.01.

## 4 Discussion

The current study shows that HCD leads to swelling and enlargement of the foam in the rumen, which hinders the expulsion of rumen fermentation gases, thus leading to rumen bloat (31). In the present experiment, a large amount of foam was also observed during the collection of rumen fluid from highly grouped goats. There is a lack of research on the causes of rumen foam formation, our study examines factors affecting rumen foam formation and foam stability with the aim of reducing rumen bloat in ruminant production through nutritional modulation.

### 4.1 PH value and rumen foam

Previous studies have shown that when ruminants were fed HCD, the pH value in the rumen usually decreases (32–35) due to the accumulation of VFAs produced by fermentation of rumen microorganisms (36, 37). This was also verified in the present experiment. The pH value of all the samples tested in this study was below 6.5, and it was also found that the pH value of the rumen fluid was significantly negatively correlated with the foaming production and foam persistence (*P* < 0.05) (Table 2), which may be due to the fact that pH affects the hydrophilicity of the headgroups of the solution, leading to structural changes in the film of the foam fluid (38). This result suggests that the lower the rumen pH value, the higher risk of developing foamy rumen bloat, which is consistent with the higher incidence of rumen bloat in production with higher ratio of dietary concentrate.

### 4.2 Viscosity and rumen foam

The viscosity of the rumen fluid was significantly positively correlated with the foaming production and foam persistence of the goats fed the HCD (*P* < 0.05) (Table 7). Previous studies have shown that the higher the viscosity of a liquid, the more stable the foam it produces (39). Lewis et al. (40) also showed that improving liquid viscosity has a positive impact on the stability of liquid foam, which is consistent with the results of this study. Cheng et al. (41) believed that a soluble mucopolysaccharide secreted by bacteria in the rumen increased the viscosity of rumen fluid. This soluble mucopolysaccharide was an exopolysaccharide produced by microbial fermentation. However, in this study, the high viscosity of rumen fluid may also be related to the high proportion of corn, meal and cake material in the diet, because these feedstuffs also contain a high proportion of soluble non-starch polysaccharides (such as β-glucan, arabinoxylan, etc). Dissolution of endosperm cell wall macromolecules NSP in high concentrate feeds alters the molecular chain length, leading to an increase in viscosity (42). The viscosity of rumen fluid is elevated by the increase in NSP content in HCD, resulting in a large amount of stable foam in the rumen. Previous studies have shown that NSP enzymes are able to cleave the long chain structure of NSP (43), which reduces the viscosity of chowders in the gastrointestinal tract of animals. This provides a new idea to reduce the occurrence of rumen bloat by altering the viscosity of rumen fluid.

### 4.3 Protein and rumen foam

The present study found that the protein content in the RFL was much higher than that in the ROL and RRL (*P*<0.05), which indicated that the protein could be enriched in the rumen foam, which was consistent with the study of Ying et al. (44). As the skeleton component of foam, protein plays a vital role in maintaining the stability of rumen foam. Proteins are low-activity surfactants that can be adsorbed at the air-liquid interface and be carried into the foam, where they are finally enriched in the foam liquid. When its peptide chain is stretched on the foam liquid surface, it will form a two-dimensional protective network through the interaction of intramolecular and intermolecular forces, which can maintain the stability of the foam. Since true protein in ruminant saliva is almost negligible, rumen protein can only be derived from dietary and microbial sources. Although rumen microorganisms can synthesize a large number of bacterial proteins every day, there is no clue that rumen foam formation is related to bacterial proteins so far. Rumen microorganisms can also synthesize a large number of secreted proteins. If the formation of rumen foams is related to the secreted proteins of microorganisms, and only high-concentrate diet can cause the formation of a large number of rumen foams, it can be inferred that high-concentrate diet may affect the microbial community structure of the type or quantity of secreted proteins by changing the structure and composition of rumen microorganisms, and ultimately induce rumen foams. Whether this conjecture is correct remains to be proved. However, previous studies have shown that soluble protein level in feed plays a role in rumen bloating, and reducing soluble protein level in feed can reduce the risk of rumen bloating (45). The application of some antinutritional factors can reduce soluble proteins in the rumen, for example, water-soluble condensed tannins can bind to soluble proteins and convert them into condensed tannin-bound forms of proteins, thus reducing the amount of soluble proteins in the rumen and reducing the production of rumen foam (46). The key to reducing the incidence of rumen bloat is to control the amount of soluble protein in the rumen, which requires research into better nutritional strategies to counteract it.

### 4.4 Total saccharides and rumen foam

In this experiment, although the total saccharide content in the rumen fluid has a certain facilitating effect on improving the foaming production and foam persistence of the rumen fluid, the total saccharide is not significantly enriched in the foam fluid, but is more uniformly dispersed in the RFL, RRL and ROL, this is the same result as Michael et al. (44). Generally speaking, during the formation of foam, substances in the liquid that are conducive to foaming will be adsorbed on the surface of the foam, such as protein, which will change the surface activity of the foam and affect the ability of the foam to drain liquid, thus affecting the foam stability. The reason why carbohydrates could not be enriched in foam fluid may be that they are low polar molecules and thus cannot be adsorbed. Soluble polysaccharides have a high characteristic viscosity, which when dissolved in water leads to an increase in solution viscosity (47), and this increase in viscosity slows down the liquid flow rate at the surface of the foam, thus contributing to the stabilization of the foam. Previous studies have found that rumen microbial mucopolysaccharides produced by rumen bacterial fermentation are the main substances leading to the increase in rumen viscosity, and that such mucopolysaccharides may contribute to the generation of rumen foams, but this role has not been studied yet (31).

### 4.5 Minerals and rumen foam

Mineral elements play a very important role in maintaining the normal physiological activities of animals (48–51). For example, the distribution of Ca in extracellular fluid and soft tissue is related to the blood agglutination, membrane permeability, muscle contraction, nerve impulse conduction, secretion of some hormones, and activity and stability of some enzymes (49). K is the main cation in the intracellular fluid, which participates in the physiological activities of maintaining osmotic pressure to regulate acid–base and water balance. Fe is an essential component of many proteins related to the transport and utilization of oxygen. These proteins include hemoglobin, myoglobin, and many cytochromes and iron sulfur proteins in the electron transport chain. Fe is also a component or activator of several mammalian enzymes (49–51) Therefore, macro and trace mineral elements are generally supplemented in ruminant feed. This experiment found that mineral elements also have a significant impact on the foaming performance of rumen fluid. Compared with anions, mineral cations have a greater impact on the foaming performance of rumen fluid. The contents of Ni, Mg, Ca and K in the rumen fluid detected in this test, as well as the sum of cations, were significantly positively correlated with the foaming production and foam persistence of the rumen fluid (*P*<0.05) (Table 7). Harris et al. (17) reported that trace amounts of mineral cations and mineral hydroxides would affect the foam stability in some cases, the concentration of Ni^2+^ would affect the stability of rumen foam, and the reduction of foam stability was accompanied by the decrease of Ni^2+^ concentration in the foam. Smith et al. (52) found that Ca^2+^ or Mg^2+^ solution was used to spray alfalfa foliage. After eating the alfalfa, the incidence of rumen bloat of lambs increased significantly, and the concentrations of Ca^2+^ and Mg^2+^ of animals with bloat increased significantly increased. Majak et al. (53) found that the occurrence of bloat in cattle was related to the increase and decrease of K^+^ and Na^+^ concentrations in the rumen. The above research results were consistent with the results of this experiment. It is worth noting that, unlike other cations, Na^+^ showed a significant negative correlation with the foaming performance of rumen fluid in this study, suggesting that increasing the concentration of Na in HCD may significantly reduce the occurrence of rumen bloat Rate. This finding can be used in the development of technologies to prevent high concentrate diet rumen bloat.

Rumen bloat can have a direct impact on animal health, which in turn can lead to economic losses. In studies by Rumbaugh (54) and Tanner et al. (55), the average annual loss due to rumen bloat in Australia was estimated at $180 million, while in the United States it was estimated at more than $310 million per year. These figures underscore the financial strain imposed on livestock production due to rumen bloat. It is vital to find out what is causing the bloat. In this study, the influence of various components of rumen fluid on the interrelationship of foam formation was analyzed in depth. Correlation analyses emphasized the important relationship between protein, total sugars and mineral elements and rumen fluid foaming characteristics, but the partial correlation analysis showed that after controlling the influence of other factors, only protein was still related to the foam persistence of rumen foam. This suggests that, although a variety of components contribute to foaming, the production and stability of foam in rumen fluid is primarily influenced by the presence and impact of proteins. This finding confirms the conclusions of Isabel et al. (56). This demonstrates the decisive role of proteins in all the factors affecting the foaming properties of rumen liquor. Tannin is a secondary metabolite in plants. Tannin can promote protein metabolism in the rumen of ruminants and increase the absorption of amino acids in the small intestine of animals. Some studies have shown that adding tannins to diets can eliminate bloat, due to the fact that tannins can destroy protein foam (57). However, tannins are poorly palatable and have a bitter taste that animals do not like to feed on. In one study, goats and sheep preferred poorly palatable hay when fed high grain concentrates, and the addition of plant odors to feed increased ruminant intake (58). This is feasible for the addition of moderate amounts of tannins to feeds in production to control rumen bloat. In addition, the defoamer’s help to break the tension on the surface of the bubbles, thus making it easier for the bubbles to burst. Defoamers accelerate the fusion of bubbles, making it easier for gases to escape. Such additives require further research.

This study highlights the importance of proteins in influencing rumen foam performance, but further research is needed to more fully understand the role of other factors in rumen distension. For example, total saccharide, its contribution to the foaming performance of rumen fluid may be because it increases the viscosity of the whole rumen fluid system, enriches more protein into the foam of rumen fluid, and helps to improve the stability of rumen foam. Sodium bicarbonate was added to the diets in this study, which is commonly used in production to increase rumen pH and reduce the risk of rumen acidosis, but sodium bicarbonate and other inorganic salts in the feeds react with fatty acids to form carboxylates (59), which have a better foaming function. Does this have an effect on rumen foam formation, Whether this could have an effect on rumen foam formation needs further investigation. Additionally, although this study showed that proteins are the main factor influencing rumen foam production and foam stability, no in-depth study was conducted to investigate the source of the proteins. A deeper understanding of the function of proteins in the rumen and their association with foam formation can help improve feed formulation and management practices to reduce the incidence of rumen foamy bloat.

## 5 Conclusion

This study found that protein content in rumen fluid was significantly and positively correlated with foam production and foam stability under high concentrate feeding conditions in goats and played a decisive role. Reducing the protein content of the diet during production is essential for rumen bloat prevention. Application of low protein diets is an effective measure to prevent rumen bloat.

## Data availability statement

The original contributions presented in the study are included in the article/Supplementary material, further inquiries can be directed to the corresponding author.

## Author contributions

ZT: Writing – original draft, Writing – review & editing. LW: Formal analysis, Funding acquisition, Supervision, Writing – review & editing. JFL: Project administration.
