# Supplementary material for: Factors affecting the rumen fluid foaming performance in goat fed high concentrate diet
Source: Front Vet Sci. 2024 Feb 16;11:1299404. doi: 10.3389/fvets.2024.1299404 (PMC10904640; doi:10.3389/fvets.2024.1299404)
Supplement: Supplementary file 1 [file Data_Sheet_1.docx]

Supplementary Material

# Supplementary Data

Day14

| **Group** | **Score** |
| --- | --- |
| 1 | 0 |
| 2 | 1 |
| 3 | 3 |
| 4 | 2 |
| 5 | 1 |
| 6 | 3 |
| 7 | 0 |
| 8 | 1 |
| 9 | 0 |
| 10 | 2 |
| 11 | 1 |
| 12 | 0 |
| 13 | 3 |
| 14 | 3 |
| 15 | 3 |
| 16 | 1 |
| 17 | 0 |
| 18 | 0 |
| 19 | 1 |
| 20 | 0 |
| 21 | 1 |
| 22 | 0 |
| 23 | 2 |
| 24 | 0 |
| 25 | 3 |
| 26 | 2 |

Day15

| **Group** | **Score** |
| --- | --- |
| 1 | 0 |
| 2 | 2 |
| 3 | 3 |
| 4 | 1 |
| 5 | 1 |
| 6 | 3 |
| 7 | 2 |
| 8 | 0 |
| 9 | 0 |
| 10 | 0 |
| 11 | 2 |
| 12 | 1 |
| 13 | 3 |
| 14 | 3 |
| 15 | 3 |
| 16 | 1 |
| 17 | 0 |
| 18 | 0 |
| 19 | 1 |
| 20 | 0 |
| 21 | 0 |
| 22 | 0 |
| 23 | 1 |
| 24 | 0 |
| 25 | 3 |
| 26 | 3 |

Day16

| **Group** | **Score** |
| --- | --- |
| 1 | 0 |
| 2 | 1 |
| 3 | 3 |
| 4 | 2 |
| 5 | 2 |
| 6 | 3 |
| 7 | 2 |
| 8 | 2 |
| 9 | 0 |
| 10 | 2 |
| 11 | 1 |
| 12 | 0 |
| 13 | 3 |
| 14 | 3 |
| 15 | 3 |
| 16 | 1 |
| 17 | 0 |
| 18 | 3 |
| 19 | 1 |
| 20 | 0 |
| 21 | 2 |
| 22 | 0 |
| 23 | 1 |
| 24 | 0 |
| 25 | 3 |
| 26 | 2 |

Day17

| **Group** | **Score** |
| --- | --- |
| 1 | 0 |
| 2 | 3 |
| 3 | 3 |
| 4 | 1 |
| 5 | 2 |
| 6 | 3 |
| 7 | 1 |
| 8 | 2 |
| 9 | 0 |
| 10 | 1 |
| 11 | 2 |
| 12 | 1 |
| 13 | 3 |
| 14 | 3 |
| 15 | 3 |
| 16 | 2 |
| 17 | 0 |
| 18 | 2 |
| 19 | 2 |
| 20 | 0 |
| 21 | 2 |
| 22 | 0 |
| 23 | 2 |
| 24 | 0 |
| 25 | 3 |
| 26 | 3 |

Day18

| **Group** | **Score** |
| --- | --- |
| 1 | 0 |
| 2 | 2 |
| 3 | 3 |
| 4 | 1 |
| 5 | 2 |
| 6 | 3 |
| 7 | 1 |
| 8 | 1 |
| 9 | 0 |
| 10 | 2 |
| 11 | 2 |
| 12 | 1 |
| 13 | 3 |
| 14 | 3 |
| 15 | 3 |
| 16 | 2 |
| 17 | 0 |
| 18 | 2 |
| 19 | 2 |
| 20 | 0 |
| 21 | 3 |
| 22 | 0 |
| 23 | 1 |
| 24 | 0 |
| 25 | 3 |
| 26 | 2 |

Day19

| **Group** | **Score** |
| --- | --- |
| 1 | 0 |
| 2 | 2 |
| 3 | 3 |
| 4 | 2 |
| 5 | 2 |
| 6 | 3 |
| 7 | 1 |
| 8 | 2 |
| 9 | 0 |
| 10 | 1 |
| 11 | 1 |
| 12 | 0 |
| 13 | 3 |
| 14 | 3 |
| 15 | 3 |
| 16 | 0 |
| 17 | 0 |
| 18 | 1 |
| 19 | 2 |
| 20 | 0 |
| 21 | 2 |
| 22 | 0 |
| 23 | 1 |
| 24 | 0 |
| 25 | 3 |
| 26 | 2 |
